# Investigating the origins of the mutational signatures in cancer

**DOI:** 10.1093/nar/gkae1303

**Published:** 2025-01-08

**Authors:** Gunnar Boysen, Ludmil B Alexandrov, Raheleh Rahbari, Intawat Nookaew, Dave Ussery, Mu-Rong Chao, Chiung-Wen Hu, Marcus S Cooke

**Affiliations:** Department of Environmental Health Science, University of Arkansas for Medical Sciences, 4301 West Markham St, Little Rock, AR 72205, USA; The Winthrop P Rockefeller Cancer Institute, University of Arkansas for Medical Sciences, 4301 West Markham St, Little Rock, AR 72205, USA; Department of Cellular and Molecular Medicine, University of California San Diego, 9500 Gilman Dr, La Jolla, CA 92093, USA; Cancer, Ageing and Somatic Mutation (CASM), Wellcome Trust Sanger Institute, Wellcome Genome Campus, Hinxton, Cambridge CB10 1SA, UK; Department of BioMedical Informatics, The University of Arkansas for Medical Sciences, 4301 West Markham St, Little Rock, AR 72205, USA; Department of BioMedical Informatics, The University of Arkansas for Medical Sciences, 4301 West Markham St, Little Rock, AR 72205, USA; Department of Occupational Safety and Health, Chung Shan Medical University, Jianguo N Rd, South District, Taichung 40201, Taiwan; Department of Occupational Medicine, Chung Shan Medical University Hospital, Jianguo N Rd, South District, Taichung 40201, Taiwan; Department of Public Health, Chung Shan Medical University, Jianguo N Rd, South District, Taichung 40201, Taiwan; Oxidative Stress Group, Department of Molecular Biosciences, University of South Florida, 4202 E. Fowler Avenue, Tampa, FL 33620, USA; Cancer Biology and Evolution Program, H. Lee Moffitt Cancer Center and Research Institute, 4202 E. Fowler Avenue, Tampa, FL 33612, USA

## Abstract

Most of the risk factors associated with chronic and complex diseases, such as cancer, stem from exogenous and endogenous exposures experienced throughout an individual’s life, collectively known as the exposome. These exposures can modify DNA, which can subsequently lead to the somatic mutations found in all normal and tumor tissues. Understanding the precise origins of specific somatic mutations has been challenging due to multitude of DNA adducts (i.e. the DNA adductome) and their diverse positions within the genome. Thus far, this limitation has prevented researchers from precisely linking exposures to DNA adducts and DNA adducts to subsequent mutational outcomes. Indeed, many common mutations observed in human cancers appear to originate from error-prone endogenous processes. Consequently, it remains unclear whether these mutations result from exposure-induced DNA adducts, or arise indirectly from endogenous processes or are a combination of both. In this review, we summarize approaches that aim to bridge our understanding of the mechanism by which exposure leads to DNA damage and then to mutation and highlight some of the remaining challenges and shortcomings to fully supporting this paradigm. We emphasize the need to integrate cellular DNA adductomics, long read-based mapping, single-molecule duplex sequencing of native DNA molecules and advanced computational analysis. This proposed holistic approach aims to unveil the causal connections between key DNA modifications and the mutational landscape, whether they originate from external exposures, internal processes or a combination of both, thereby addressing key questions in cancer biology.

## Graphical Abstract

**Graphical Abstract:**
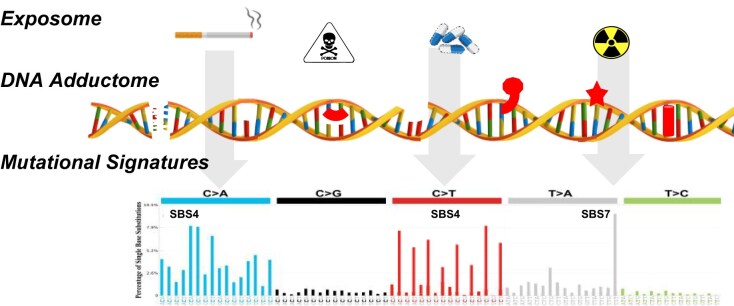

## Introduction

### Mutational signatures

#### Introducing mutational signatures and landscapes

Advances in DNA sequencing have drastically enhanced our knowledge about the genetic changes that occur during the development and evolution of tumors (1–3). Characteristic mutational signatures are observed by analyzing the patterns of mutations across the genome. While there are only six basic types of single base substitutions (SBSs) (C>A, C>G, C>T, T>A, T>C and T>G; substitutions referred to by the pyrimidine of the mutated Watson–Crick base pair), the context in which these mutations occur can vary significantly. This context is determined by the nucleotides flanking the mutated DNA base on either side. For each mutation type, the identity of the nucleotides immediately before and after the mutated nucleobase can influence the likelihood of that mutation occurring (4). By examining large catalogs of somatic mutations from various samples, recurring patterns of mutations are identified within specific nucleotide contexts. These patterns, or mutational signatures, provide insights into the underlying mechanisms causing the mutations. For example, a C>T mutation might be more frequent when the cytosine is preceded by a thymine (T) and followed by a guanine (G), forming a specific trinucleotide context (e.g. TpCpG; mutated base underlined). Another mutational signature might involve a different context, such as an A preceding the C and a T following it (e.g. ApCpT). By cataloging these mutational signatures within a trinucleotide context, specific patterns can be linked to different mutational processes, such as exposure to certain carcinogens, defects in DNA repair pathways or the activity of endogenous enzymes such as APOBEC enzymes or DNA polymerases (5). This detailed understanding allows for more precise identification of the mutagenic factors involved in cancer and other diseases, ultimately contributing to better diagnostic, preventive and therapeutic strategies.

The genomic landscape of somatic mutations across many thousands of tumors and healthy tissues has been curated in the Catalogue of Somatic Mutations in Cancer (COSMIC) database, a publicly available repository that provides annotation tools for data mining, analysis and visualization (6). Furthermore, the Cancer Gene Census is an ongoing effort to catalog those genes that contain mutations that have been causally implicated in cancer and explain how dysfunction of these genes drives cancer (7). The Cancer Mutation Census (CMC) project is an undertaking to classify coding mutations in COSMIC and identify variants driving diverse types of cancer. The CMC integrates all coding somatic mutations collected by COSMIC with biological and biochemical information from multiple sources, combining data obtained from manual curation and computational analyses.

Comprehensive analyses employing whole-genome and whole-exome sequencing data have revealed a diverse and complex landscape of mutational signatures across human normal tissues (8) and cancers (2). The patterns of somatic mutations found in the genomes of both normal and cancerous tissues reflect a complex interplay of multiple mutational processes, each active at different stages of an individual’s life and contributing distinct numbers of mutations (2). Using unsupervised machine learning techniques, such as non-negative matrix factorization, we demonstrated that one can identify the operative mutational signatures within a set of cancer genomes, along with the number of mutations each signature contributes to each cancer sample (2). Ideally, a cancer genome’s mutational pattern is considered well-explained when the cosine similarity between the reconstructed mutation profile (derived from identified mutational signatures and their contributions) and the original mutation profile is at least 0.90. This approach has subsequently been utilized to examine the mutational signatures in many thousands of cancer genomes (9,10).

**Figure 1.**
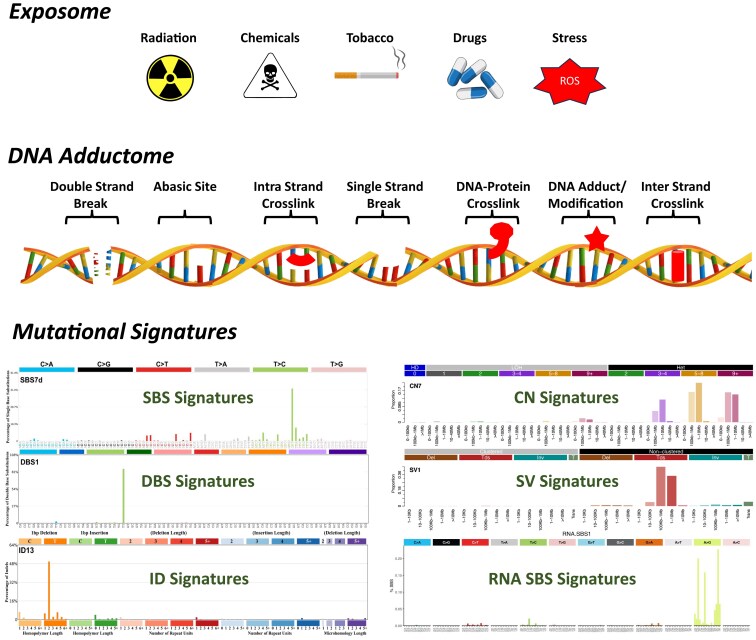
The exposome encompasses the totality of human environmental (both endogenous and exogenous) exposures across the lifespan. The exposome represents a complex mixture of multiple, diverse exposures many of which can directly or indirectly modify, or damage DNA. These DNA modifications can lead to various types of somatic mutations, including SBSs, doublet base substitutions (DBSs), small insertions and deletions (IDs), copy number changes (CNs) and structural variations (SVs). SBSs involve the replacement of one nucleotide with another and are among the most common mutations in cancer. DBSs refer to simultaneous substitutions of two adjacent bases. IDs involve the addition or loss of small DNA segments, potentially disrupting gene function. CN changes involve gains or losses of larger DNA regions, leading to gene amplification or deletion. SVs encompass large-scale genomic alterations, such as deletions, duplications, inversions and translocations, which can disrupt gene structure and regulatory regions, contributing to cancer progression.

A recent groundbreaking study by the International Cancer Genome Consortium, The Cancer Genome Atlas and Pan-Cancer Analysis of Whole Genomes Network identified these mutational signatures utilizing data from over 23 000 cancer patients (6). With an enhanced understanding of somatic mutation prevalence in different normal tissues and tumors, concerted efforts have been made to identify and classify mutational signatures that could pinpoint the mutagenic origins and etiology of cancers to inform future cancer prevention and intervention strategies (8) (Figure 1).

Characteristic mutational signatures have been defined based on distinct types of somatic mutations (11). SBS signatures are defined as the replacement of a certain base pair with a dissimilar one (12). DBSs arise after the concurrent modification of two consecutive nucleobases (6). Small ID signatures, also known as indels, are defined as the incorporation or loss of small fragments of DNA (usually between 1 and <50 bp) at a specific genomic location (13). CN signatures are common in many cancers (14,15) and are distinguished by large genomic segments with alterations in the number of DNA copies (16). SV signatures are based on large genomic changes, typically exceeding 1 kb in length, which affect the arrangement and structure of the genome (17). Thus far, almost 200 characteristic signatures have been identified, comprising of at least 99 SBSs, 20 DBSs, 23 IDs, 25 CNs and 10 SVs (6,16,18). Some mutational signatures have been associated with specific exogenous chemical exposures, e.g. SBS4, which is likely due to direct DNA damage by tobacco smoke mutagens (11) and often associated with ID3, and DBS2 (19,20). While some have an endogenous origin, e.g. SBS1, a clock-like signature, which is thought to result from the spontaneous or enzymatic deamination of 5-methylcytosine (5-Me-Cyt) to thymine, leading to mismatched Gua in double-stranded DNA (21), others are results of deficiencies in DNA repair systems, e.g. SBS6, error-prone DNA replication or other cellular processes (2,8). Additionally, several mutational signatures classified as known or possible artifacts have been identified. It is important to note that COSMIC employs a conservative approach in assigning etiologies (6). For instance, mutational signatures labeled as possible artifacts may indeed represent genuine mutational processes, but they are designated as such because they often originate from single cohorts with small sample sizes, limiting their validation and broader acceptance. Conversely, while many signatures with unknown etiologies lack detailed mechanistic characterization, studies have suggested potential associations for some of these signatures. However, these associations have not yet reached the level of robust validation needed to establish a definitive etiology. Excluding known and possible sequencing artifacts, there are 146 total mutational signatures in COSMIC, of which 64 have unknown etiologies, highlighting the significant gaps that remain in understanding the origins of these (Supplemental Table S1). For a comprehensive overview of the nearly 200 established mutational signatures, including ones attributed to known and possible sequencing artifacts, readers are encouraged to visit the COSMIC database (https://cancer.sanger.ac.uk/cosmic/signatures), which offers extensive information and tools for navigating these data.

#### Mechanisms of mutagenesis

At its simplest, DNA mutations arise due to error-prone DNA replication, or the replication of DNA following incorrect repair of DNA lesions. Mechanistically, mutations arise when polymerases erroneously replicate DNA, with error rates significantly escalating when encountering DNA modifications/adducts. To clarify, for this review, DNA modifications encompass (i) DNA adducts, some of which serve as well-established biomarkers of exposures and precursors of mutagenesis, (22–24), (ii) DNA adducts derived from endogenous reactions or DNA degradation (25,26) and (iii) epigenetic marks that are added by cellular processes (27).

Site-directed mutagenesis studies with individual DNA adducts demonstrate that mutational signatures of DNA adducts are directly related to the DNA polymerase(s) that bypass them, together with the mechanism of their nucleotide insertion and extension, as well as the DNA sequence context (28).

Under normal conditions DNA can be replicated with high accuracy, with estimated error rates as low as one error in a billion bases replicated (i.e. one error per 10^9^ bp) (29). This remarkable accuracy is achieved by highly redundant repair mechanisms acting in concert and with varying error rates (29): base selection (one error per 10^4^–10^5^ bp); 3′ to 5′ exonuclease proofreading (one error per 10^2^ bp); and mismatch repair (following replication; 10^−3^). Although error rates can vary more than a million-fold, depending on cell types and stage, the DNA adduct and the sequences being replicated (30). Even this highly accurate system accumulates mutations over time in normal tissues, leading to the emergence of two predominant mutational signatures known as aging signatures (SBS1 and SBS5), which are pervasive across nearly all tissues (31).

In genomic DNA, the presence of DNA adducts during cell division can impede the progression of the replication fork, culminating in cell death. To circumvent this fate, cells deploy mechanisms to navigate past these obstacles. Two primary pathways facilitate DNA replication bypass: (i) damage avoidance strategies such as replication fork regression and recombination repair, which is an error-free mechanism, and (ii) translesion synthesis (TLS), which can be either error-free or error-prone depending on the structural characteristics of the DNA adduct and the DNA polymerases involved (32,33).

DNA polymerases that specialize in TLS often lack proofreading activity such as 3′ to 5′ exonuclease activity (34). Consequently, these TLS polymerases have a predisposition to inserting an incorrect nucleotide opposite the DNA adduct, potentially leading to base-change mutations. The error rates of the TLS polymerases are in the range of 0.001–100% and depend on the nature of DNA adduct to be bypassed and its flanking sequence context (33). Incorporating the overarching mechanisms governing the efficiency, propensity for errors and the involvement of specific polymerases in TLS of DNA modifications or DNA adducts is a requisite to the identification of the etiologies of mutational signatures. The current paradigm for TLS is that when a processive DNA polymerase encounters a blocking DNA modification, the polymerase dissociates, and a TLS polymerase binds to the DNA, incorporating a 2′-deoxyribonucleoside triphosphate opposite the modified nucleobase, reviewed in Basu *et al.* (33).

#### Mutational signatures are the result of chemical exposures and cellular stressors

**Figure 2.**
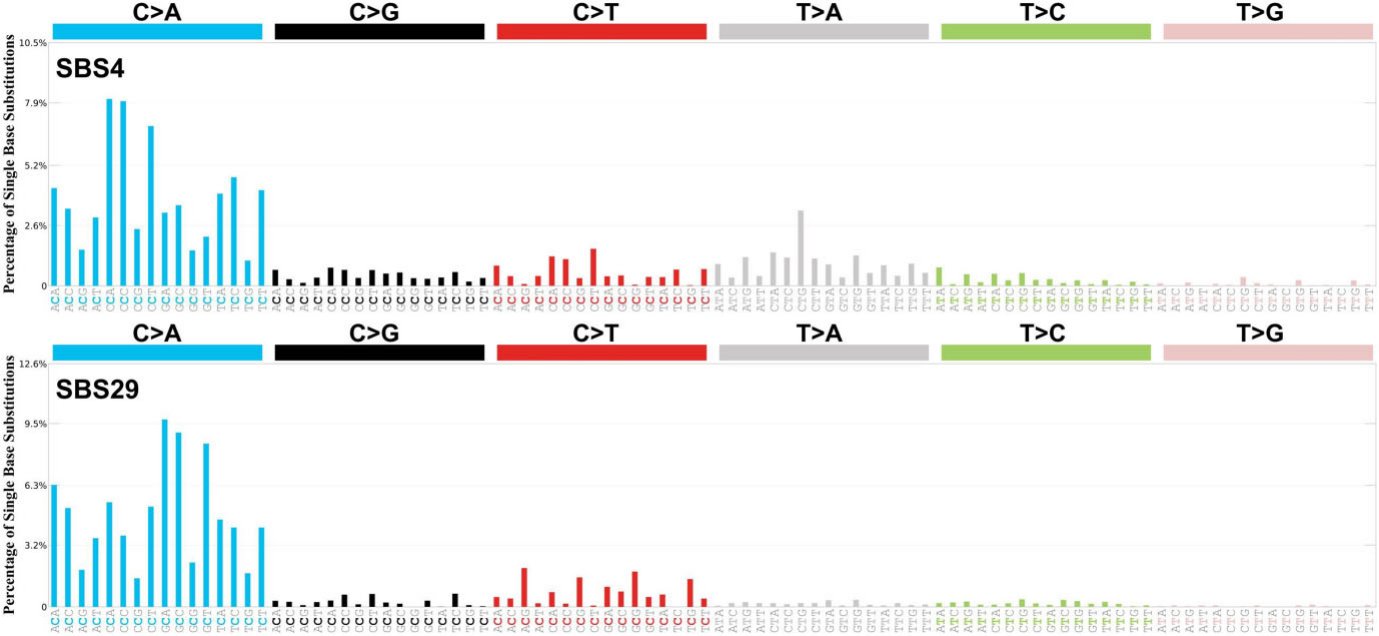
The representative mutational signatures, SBS4 and SBS29, are unique enough to be distinguished from others, associated with a specific exposure and are found in the corresponding tumors. SBS4 (top panel) is associated with tobacco use and found primarily in tumors from tobacco smokers (35), while SBS29 (bottom panel) is associated with chewing tobacco and almost exclusively in oral cancers. Both exposures lead to tumors with predominantly C > A mutations; however, the trinucleotide context profile suggests distinct differences in the mutational signatures, and hence perhaps mechanisms, induced by smoking tobacco compared with those induced by chewing tobacco, which could be elucidated by studying of the DNA adductome.

Epidemiologic studies demonstrate that environmental exposures are responsible for as much as 80–90% of the risk of developing cancer and other diseases (35,36), linking disease risk to components of the exposome, which encompasses all internal and external environmental factors to which a human is exposed over the lifetime (37). However, identifying the specific exposures responsible and then linking them to mutational signatures is challenging. The COSMIC consortium applies stringent and conservative approach when assigning etiologies to mutational signatures (2,5,6). For example, SBS4 is enriched in lung cancers of tobacco smokers when compared with lung cancers of never smokers. Moreover, the number of SBS4 mutations correlates with the pack-years smoked reported by lung cancer patients (dose-response). Additionally, these associations have been confirmed over multiple cohorts of lung cancer as well as other cancer types, which makes them more reliable (i.e. not an coincidence from a specific set of patients or cohorts) (38). As a result, there are multiple lines of evidence suggesting causal link between tobacco use and SBS4 which is primarily found in normal lung tissues of former and current smokers (20) and lung, head and neck and esophagus tumors from tobacco smokers (39) while SBS29 is associated with chewing tobacco and almost exclusively observed in oral cancers (40) (Figure 2). Further, SBS22 and SBS24 have been found in pre-cancerous tissues and tumors derived from exposures to aristolochic acid (41) and aflatoxin, respectively (42). These mutational signatures are clearly linked to known chemical exposures and tissue-specific tumorigenesis.

**Figure 3.**
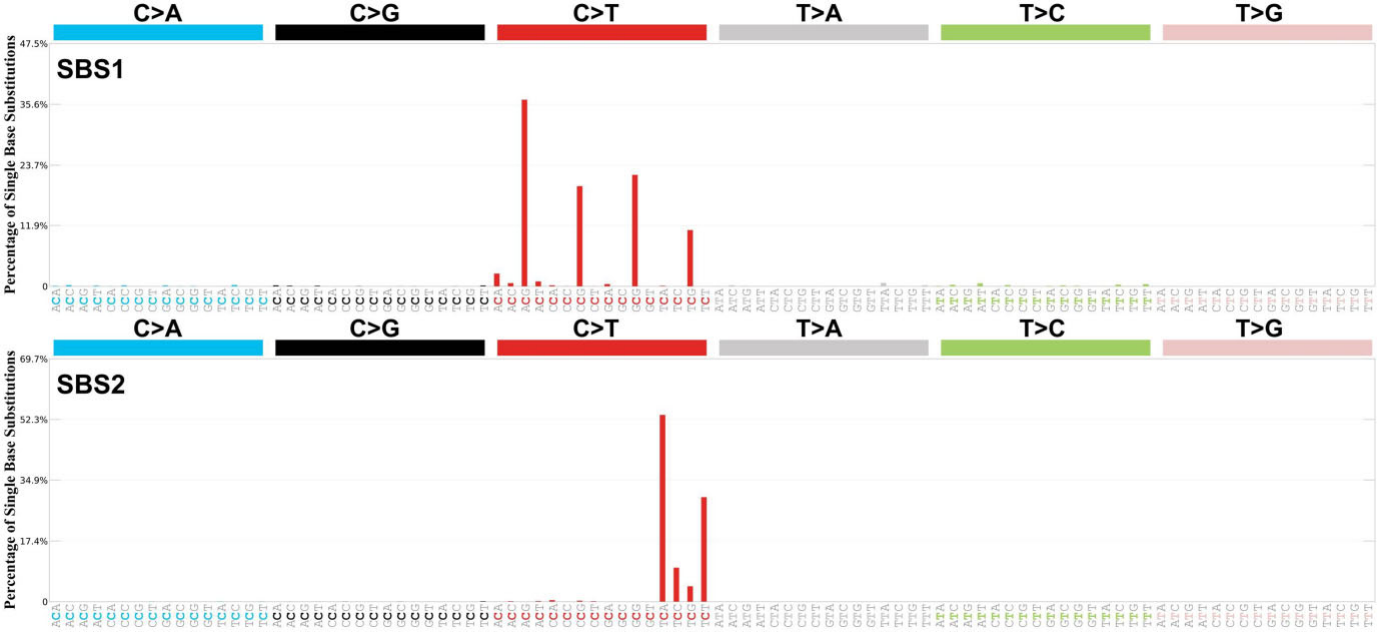
Representative mutational signatures, SBS1 and SBS2, are distinct enough to be distinguished from others and are associated with specific endogenous processes. SBS1 (top panel) is thought to result from the spontaneous or enzymatic deamination of 5-Me-Cyt to Thy, while SBS2 (bottom panel) is associated with cytidine deaminase activity, specifically the AID/APOBEC family of cytidine deaminases. Both endogenous processes lead to mutational signatures characterized by C>T mutations; however, each exhibits a unique trinucleotide context profile, suggesting different underlying mechanisms.

In contrast to environmentally induced mutational signatures, SBS1 and SBS5 appear to be a modification of endogenous origin, seemingly arising due to spontaneous deamination of 5-Me-Cyt (2,43), and are found to accumulate with age in all normal somatic tissues of the humans and the mammals studied to date (8,21). SBS1 is characterized by C>T transition mutations and is believed to result from errors in epigenetic remodeling, such as the addition and removal of 5-Me-Cyt (44). In contrast, SBS2, while also dominated by C>T transition mutations, has a distinct trinucleotide context profile and is associated with cytidine deaminase activity, particularly from the AID/APOBEC family of cytidine deaminases (Figure 3).

Other signatures are linked to defective DNA repair systems (e.g. SBS3, SBS6, SBS15, SBS20 and SBS26) (6,45,46) or DNA metabolism involving enzymes such as 5-methylcytidine deaminase, or errors during DNA replication, e.g. arising from a faulty error-prone polymerase (e.g. SBS10a/b/c/d). However, for the majority of mutational signatures their etiology remains unknown, severely limiting our ability to dissect the origins of cancer and develop interventions.

## Considerations when identifying the origin of mutational signatures

To aid in the identification of the etiology of mutational signatures, there is an urgent need to evaluate the molecular mechanisms associated with the formation of each signature with, yet, unknown etiology.

First, it is important to understand that the mutations found in tumor genomes are biased as tumorigenesis selects for DNA changes that maintain or accelerate cellular growth. Therefore, mutational signatures will not include somatic mutations where DNA adducts have formed and were repaired correctly prior to replication, or where DNA adducts and/or somatic mutations have led to cell death (10,47–51). Consequently, determining the etiology of a given mutational signature will depend on a fraction of the initial induced DNA damage that had not been repaired and led to mutations – these mutations are the ‘ripples’ which are observable in the cancer genome.

Second, DNA is a dynamic molecule, which is constantly being modified by various endogenous processes (52). Amongst the most prominent endogenous processes are the epigenetic remodeling processes that respond to cellular environmental factors. Epigenetic regulation involves writing, reading and erasing epigenetic DNA marks depending on the status and needs of the cells (53). In this review, we will refer to this process as a form of DNA modification distinct from DNA adducts, since alkyl-groups are enzymatically attached and actively removed by cellular processes essential for cell survival. The most common DNA modification is 5-Me-Cyt, followed by 6-methyl-adenine (6-Me-Ade) (54). Furthermore, the levels of 5-Me-Cyt, and its derivatives, can form an epigenetic timeline where 5-Me-Cyt can be oxidized to 5-hydroxymethylcytosine (5-hMe-Cyt); this can be further oxidized to 5-formylcytosine (5-f-Cyt) and 5-carboxylcytosine (5-CaCyt) and then back to unmodified dC (55).

Third, DNA is also modified through reactions with reactive metabolites forming covalent bonds with the nucleobases. The amount and type of DNA adducts present in DNA reflect the nature, duration and magnitude of exposure(s) and processes such as activation, detoxification and repair (56). Consequently, the exposome is comprised of multiple, distinct different types of adducts in DNA, arising from multiple complex exposures. Even a single toxicant can form multiple DNA adducts. For example, the tobacco-specific nicotine-derived nitrosamine ketone is known to form several alkyl and methyl DNA adducts (57). Other DNA adducts are formed by reactive metabolites that are generated constantly via cellular metabolism, or arise from the diet or other environmental sources. Such reactive metabolites include reactive oxygen species (ROS), reactive nitrogen species, together with reactive aldehydes and carbonyls (58–60), which have the potential to modify cellular biomolecules and hence generate DNA adducts (61). Further, DNA can be modified by spontaneous deamination, de-glycosylation and breakage of the phosphate backbone. The subsequent DNA degradation products of nucleobases, 2-deoxyribose and the phosphate backbone can cause mutations during replication (25).

Lastly, ribonucleotides are frequently incorporated into DNA during replication, making them common non-canonical nucleotides in genomic DNA (62,63). It is estimated that millions of ribonucleotides can be incorporated into the mammalian genome during each round of replication. The presence of ribonucleotides in DNA leads to genomic instability, DNA structure alteration, ultimately contributing to mutagenesis (64). Cells have evolved specific repair mechanisms, such as ribonucleotide excision repair, to remove these misincorporated ribonucleotides (65,66).

### Exposures induce DNA adducts and DNA modifications

#### The exogenous DNA adductome

**Figure 4.**
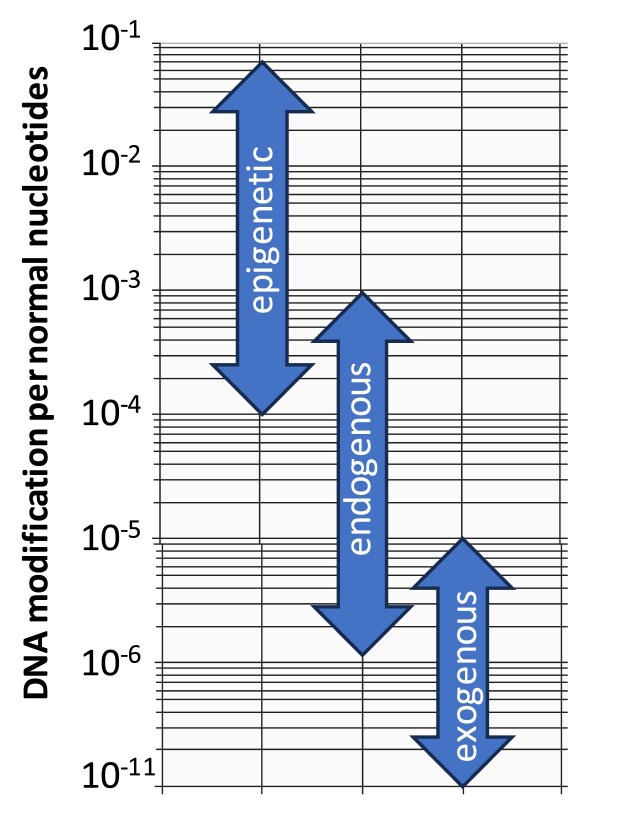
An illustration of the range of DNA adducts and DNA modifications derived from epigenetic remodeling, endogenous and exogenous sources.

Over the last century, it has been well established that humans are exposed to genotoxic, mutagenic and carcinogenic agents in the workplace and more broadly in their environment. Numerous studies have shown that many carcinogens, either directly or after metabolic activation, covalently bind to DNA, forming DNA adducts (67,68). These DNA adducts may be of endogenous (i.e. they arise without the need for any exogenous exposure) or exogenous origin (i.e. an exogenous exposure is required for these kinds of adducts to be formed). It is important to note that exogenous exposures can influence endogenous processes to give rise to adducts that are identical to the ones from endogenous origins, but not *vice versa*. Many DNA adducts derived from exogenous sources retain the chemical identity of the initial exposure, making them excellent biomarkers of internal exposures. Classic examples of exogenous DNA adducts are *N*2-[7,8,9-trihydroxy-7,8,9,10-tetrahydro-benzo[*a*]pyrene-10-yl]deoxyguanosine (*N*^2^-BPDE-dG), which is derived from benzo[*a*]pyrene, an ubiquitous occupational and environmental pollutant derived from incomplete combustion (23,69), cyclobutane pyrimidine dimers from ultraviolet (UV) radiation (70), 8,9-dihydro-8-(N7-guanyl)-9-hydroxyaflatoxin B1 from dietary aflatoxin B1 (71) and 7-(2′-deoxyadenosin-N6-yl)aristolactam I from plants containing aristolochic acid (72). Depending on the type of exposure, uptake and metabolic activation, the majority of the initial DNA adducts are removed by the various constitutive or induced DNA repair systems. However, as exposure continues exogenous DNA adducts typically reach a steady state, where their rate of formation equals their rate of removal by DNA repair, in the range of one DNA modification per 10^8^–10^11^ normal nucleotides (Figure 4).

#### The endogenous DNA adductome

As detection methods improved, DNA adducts were observed in cells or tissues that were not known to have been exposed, suggesting that DNA adducts can also arise from endogenous cellular processes. A major mechanism leading to the formation of these endogenous DNA adducts is the production of ROS, and the associated lipid peroxidation (73). In addition, many cellular processes form aldehydes and other reactive intermediates that may bind to DNA (58–61). Amongst the most common endogenous modifications detected in DNA are aldehydic modifications associated with apurinic/apyrimidinic sites, an intermediate of DNA repair process or resulting from DNA nucleobase loss due to oxidation or spontaneous depurination and depyrimidination of alkylated and normal nucleobases (26,74,75). Together a single human cell may harbor 50,000–100,000 DNA modifications just from endogenous cellular activities (25,26). Under normal conditions individual endogenous DNA adducts may reach a steady state, of one DNA adduct per 10^3^ or 10^6^ normal nucleotides (Figure 4). In contrast to exogenous DNA adducts, DNA adducts of an endogenous origin are unlikely to be unique to a particular stressor, and are invariably present at detectable levels in all cells and tissues (76).

#### Epigenetic DNA modifications

The discovery that epigenetic DNA modifications are constantly added and removed to the DNA provides another layer to the dynamic of DNA remodeling and the potential for errors and mutagenesis. An estimated 2–4% of all cytosines and 0.05–1.0% of all adenines are methylated (77–80), making these the most abundant DNA modifications in human cells with one epigenetic DNA modifications in roughly 20 normal nucleobases, or about 300 million modified nucleobases per diploid human genome, or one epigenetic DNA modification per 10^1^–10^2^ normal nucleotides (Figure 4) (81,82), reviewed in Lentini *et al.* (83). Approximately one-third of all point mutations in the human genome are C>T transitions, with the majority occurring at CpG sites. This is primarily attributed to the spontaneous deamination of 5-me-Cyt, which converts it to thymine, creating T:G mismatches that, if unrepaired, result in permanent C>T mutations. These mutations are particularly problematic because thymines pair perfectly with adenine, bypassing DNA repair mechanisms and leading to mutational hotspots in the genome. CpG dinucleotides are therefore recognized as prominent contributors to the mutational burden in cancer and other diseases (84).

Recent research by Tomkova *et al.* (85) highlights an alternative mechanism for C>T mutagenesis at CpG sites. Using polymerase error rate sequencing, the study identifies replication errors as a significant contributor. The mutant polymerase ϵ (Pol ϵ P286R) produces an excess of CpG>TpG errors, replicating the mutational patterns observed in mismatch repair-deficient tumors. Even wild-type Pol ϵ demonstrates a substantially higher error rate when replicating 5mCpG sites.

Overall, while the spontaneous deamination of 5-me-Cyt has traditionally been regarded as the primary cause of C>T mutations at CpG sites, recent evidence (85) indicates that additional factors, such as replication errors, also contribute to the C>T mutation burden. Furthermore, it is plausible that other, yet unidentified mechanisms may also influence the generation of C>T mutations at these sites.

#### Exposures can produce both endogenous and exogenous DNA modifications

Exogenous exposure may also affect endogenous processes, and influence levels of endogenously derived DNA adducts and DNA modifications. For example, exposure to non-ionizing or ionizing radiation, and metabolism of certain xenobiotics may all lead to the generation of free electrons, impact redox homeodynamics and increase levels of oxidatively modified DNA above baseline levels, e.g. 8-oxo-Gua (86). Similarly, some exogenous exposure, e.g. benzo[*a*]pyrene (87), polychlorinated biphenyls, methylmercury and organochlorine pesticides can influence methylation and other epigenetic processes, modulating the levels of these DNA modifications (88). As a result, there is a degree of overlap in the types of DNA adducts and DNA modifications stemming from both exogenous and endogenous sources. While these types of DNA adducts and DNA modifications individually are not distinct markers of a specific exposure, they could potentially contribute to creating exposure-specific patterns when combined with other forms of DNA modifications. This is especially true when considering multiple forms of DNA modifications together, offering insights into the precise mechanisms through which an exposure contributes to the DNA adductome (87).

Of the processes which induce DNA adducts, oxidative stress is a prime example of the potential to induce a multiplicity of different forms of DNA adducts and has recently been defined as a hallmark of environmental insult (89). Oxidative stress leads to the formation of over 24 types of DNA nucleobase modifications, with the total number of DNA adducts exceeding 100 when including modifications to the 2-deoxyribose sugar and phosphate backbone (90), and this number do not include DNA–DNA and DNA–protein crosslinks, or the adducts derived from secondary processes, such as lipid peroxidation (91). Combined, the total number of potential types of DNA modifications is likely to be in the high hundreds, if not greater, and of which many, if not all, have the potential to affect cellular function and contribute to mutagenesis (92).

Further, some well-known epigenetic DNA marks, such as N6-hydroxymethyladenine (93,94) are indistinguishable from exposure-induced DNA adducts since they can also arise from environmental exposures, such as formaldehyde (95), making determination of their origins and therefore their role in mutagenesis, challenging.

### The DNA adductome

To elucidate the etiologies contributing to individual mutational signatures, significant efforts are ongoing to characterize the total burden (qualitative and quantitative) of DNA modifications (adducts and epigenetic modifications) in the genomes of normal and tumor tissues. In pursuit of this objective, Kanaly *et al.* introduced the term adductome and pioneered the field of DNA adductomics, aiming to measure all the DNA modifications in the genome irrespective of their origin, whether endogenous or exogenous [Kanaly *et al.* (96) and reviewed in Balbo *et al.* (97) and more recently in Möller *et al.* (98)]. As noted above, the exposome is recognized as the principal contributor to the formation of a diverse array of DNA adducts. Interactions between the exposome and the genome have been linked to mutagenesis and the risk of developing cancer and other chronic and complex diseases through the induction of DNA modifications (35,99).

Together, these lines of research provide evidence for the fundamental premise that exposure leads to DNA adducts, that if these are not repaired correctly prior to DNA replication, induces mutations (100,101). Consequently, the mutational signatures observed in human tumors reflect, to some extent, the environmental agents to which individuals were exposed during their lifetime (5,102,103). An overall goal of these efforts is to identify the environmental agents, or stressors, responsible for the adductome that causes the mutational signatures and subsequent tumor development (92).

#### Targeted DNA adductomics

The majority of studies reported in the literature use a targeted approach to analyze DNA adducts, i.e. measuring only one or a few DNA modifications selected based upon prior knowledge of the adducts of interest (67). A well-established example of this is the measurement of DNA-derived biomarkers of oxidative stress, principally 8-oxoGua and its 2′-deoxyribonucleoside equivalent 8-oxodG, which have been studied in cellular DNA and urine since the 1990s and remain very popular, but singular, targets [reviewed in Chao 2021 (104)]. Indeed, measurement of cellular and/or urinary 8-oxoGua/8-oxodG has contributed much to our understanding of the effects of redox imbalance in health and disease, and yet a broader approach, utilizing multiple oxidation products of DNA would likely to have been much more informative. Consequently, there are growing reports in the literature of targeted adductomics, in which a limited selection (numbering in the 10s) of known adducts (i.e. the inclusion list) are studied [e.g. Carra *et al.* (105) and reviewed in Villalta and Balbo (106)]. Targeted adductomics allows information on a broader range of adducts to be gathered, while limiting the inclusion list to largely known adducts and hence the availability of isotopically labelled internal standards, or other approaches, to facilitate accurate identification.

Unfortunately, despite having provided invaluable information about the mode of action of exposure-induced genotoxicity and subsequent mutagenicity, the small number of adducts measured in targeted studies fail to consider the full range of DNA modifications within the cell, resulting in crucial information being overlooked.

#### Untargeted DNA adductomics

To comprehensively detect the widest range of potential DNA modifications simultaneously, untargeted adductomics approaches are required and are being developed, i.e. studying the totality of all DNA modifications (27,107).

The advent of high-resolution mass spectrometry (HRMS) for determining the DNA adductome has led to the proposed ‘top-down’ approach by which patterns of DNA modifications are used to trace and identify the source of the originating exposure. We and others have reported a cellular DNA adductomics approach that includes HRMS (27,95,108–111). It is important to highlight that DNA adductomics does not exclusively detect DNA adducts formed through alkylation reactions; it also identifies ‘intentional’ products, like epigenetic DNA modifications, resulting from normal cellular processes and DNA degradation products (112). Consequently, DNA adductomics can indicate, at least in part, the mode of action of a stressor and thereby inform on the mechanisms responsible for causing the mutational landscape.

Unfortunately, while HRMS-based DNA adductomics provides information as to the nature and quantity of the ‘totality’ of DNA modifications within the genome, it does not provide information concerning the location of these DNA modifications across the genome, thereby hindering the accurate assignment of a particular DNA adduct type to a site-specific mutation or a mutational signature.

#### Mapping of DNA modifications

In parallel to the DNA adductomics efforts to characterize and quantify the plethora of DNA modifications, various methods have been used with great success for genome-wide and site-specific mapping of DNA modifications, significantly extending our understanding of the topography of the genomic DNA modification landscape [reviewed in Boysen and Nookaew (113) and Amente *et al.* (114)]. Many of these methods take advantage of DNA-repair enzymes to mark and excise adduct-containing oligodeoxynucleotides, which are subsequently sequenced to locate the position of the DNA modification, while other methods use click chemistry to label DNA adducts, or DNA adduct-specific antibodies to enrich for DNA modification-containing DNA oligos, prior to sequencing [reviewed in Amente *et al.* (114)]. Unfortunately, these elegant approaches have been limited by the breadth of repair enzyme specificity, which may result in a (unidentifiable) mixture of DNA modifications being mapped (115). Additional challenges include inadequate antibody specificity, limited availability of antibodies for only certain DNA adducts and the inability to simultaneously map and differentiate between multiple adducts. Alternative chemical labeling approaches rely on the completion of chemical labeling reactions, and their restricted applicability to specific types or classes of DNA adducts. Further, most of these labeling and excision strategies yield short DNA fragments that may not always align with the genome definitively.

We and others have shown that single-molecule real-time long-read sequencing technologies can overcome the limitations of current adduct mapping approaches and that nanopore-based technologies are suitable for identifying DNA adducts and epigenetic DNA modifications in native DNA sequences from various model systems (113,116,117).

**Figure 5.**
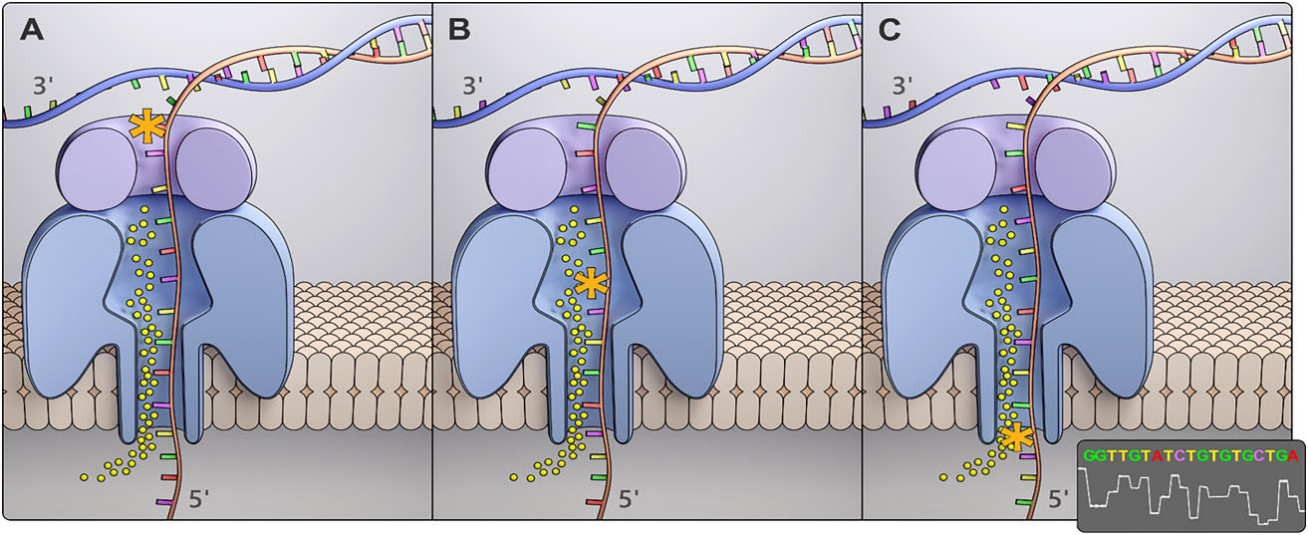
A scheme illustrating a DNA strand, containing a DNA adduct (*) passing through a nanopore and potentially blocking or altering the ion current (dots) at or near the adduct site (**A**) preceding, (**B**) at or (**C**) trailing the DNA adduct.

Nanopore technology uses electrochemical forces to pull single-stranded DNA in native form through tiny pores. The accompanying changes in electric current indicate the physicochemical properties of the DNA bases transiting through the pore, revealing the DNA sequence and identity of the DNA nucleobase, DNA modification or DNA adduct while transitioning through the pore. A DNA modification modulates the nanopore ion current signal while entering, passing through and exiting the nanopore (Figure 5). Burrows *et al.*, who pioneered this approach for sequencing DNA modifications in single-stranded DNA, showed the proof-of-principle for detecting and genome wide mapping of *N*^2^-BPDE-dG adducts (118), abasic sites (119–121), 8-oxoGua (122–124) and other DNA adducts (125). Using a similar principle, Oxford Nanopore Technologies (ONT) developed and commercialized a technology that can sequence long to ultra-long (>2 Mb) molecules of native DNA that preserves the sequence position of DNA modifications (126–128). It was recently shown that ONT could detect epigenetic modifications in DNA, such as 5-Me-Cyt and *N*6-Me-Ade, at a genome-wide scale (121,129–131). Our team developed the Epitranscriptional/Epigenomical Landscape Inferring from Glitches of ONT Signals (ELIGOS) software that simultaneously detects RNA and DNA modifications by using ONT data (113,116,132–134). The ONT/ELIGOS platform is a powerful tool for detecting DNA modifications and for discriminating DNA modifications of different sizes, regiochemistries and functional groups (133).

Furthermore, artificial intelligence (AI) based software tools are being developed for detection and localization of DNA adducts and DNA modifications (135). Some studies demonstrated the accurate performance using deep-learning model-based nanopore ionic signal to identify and localize DNA methylation in native (rather than *in silico*) sequences. Bonet *et al.* (136) developed an accurate deep-learning model and presented DeepMP software for detecting 5-Me-Cyt in nanopore native sequences. Similarly, Yin *et al.* (137) reported a hybrid deep-learning software, NanoCon, to detect 5-Me-Cyt methylated sites from nanopore reads. Ni *et al.* developed DeepSignal software that can detect 5-Me-Cyt and 6-Me-Ade methylated sites (129). Ahsan *et al.* (138) described DeepMod2, which is the upgraded version of DeepMod (121) and is able to accurately detect 5-Me-Cyt, 5-hMe-Cyt and 6-Me-Ade like the standard Oxford Nanopore base-calling software Guppy and Dorado. Together, these efforts provide proof of principle for the location-specific quantification and mapping of DNA modifications.

However, detecting DNA adducts on nanopore native sequences using a signal-based deep-learning model remains elusive (115). This is primarily due to the requirement for a substantial amount of accurate ground truth data to construct and train such a model, which is particularly challenging to obtain from routine native DNA sequencing. In contrast to DNA methylation, which is abundant in the genome and can be orthogonally validated with bisulfite sequencing, DNA adducts are rare, and orthogonal validation approaches are limited or, in many cases, non-existent. To overcome this limitation, using synthetic DNA sequences containing the adduct of interest in the desired context, could be a viable solution (133). Nonetheless, for the nanopore sequencing system, generating numerous synthetic adducted DNA sequences that accurately reflect the diverse sequence contexts and neighboring environments poses significant challenges and is likely to be a prohibitive expense.

## Remaining challenges and future directions

Whole-genome sequencing of normal and tumor tissues has advanced our understanding of the mutational processes that lead to carcinogenesis (1), but there is limited knowledge of the precise origin of the mutations which may arise from (i) exposure to exogenous/endogenous genotoxins; (ii) decreased accuracy of DNA replication; (iii) enzymatic and/or epigenetic modification of DNA; (iv) defective DNA repair or other cellular processes; (v) combinations of these; or (vi) other as yet unknown mechanisms.

A good start has been made in the identification of mutational signatures associated with environmental agents in human cancer (5,102,103). These signatures demonstrate that exposures cause genome instability and leave their imprint on the genomes of cells that persists in the resulting tumors (1). The COSMIC database currently includes 177 mutational signatures; while some, such as SBS7 linked to UV light exposure, have well-supported etiologies, less than half have clearly established causes (6,16,18). Additionally, even among those with proposed etiologies, the origins of certain signatures remain uncertain or actively debated. For example, SBS2 and SBS13 are associated with APOBEC enzymes, but the specific contributions of APOBEC3A, APOBEC3B and/or APOBEC1 are still under discussion (139–141). Notably, some of the most prevalent mutational signatures in both cancer and normal tissues, such as SBS5, SBS17 and SBS40, still lack known etiologies (6). SBS5, for instance, displays clock-like behavior (21), with mutation counts increasing with age, yet its mutational burden can vary in specific contexts, such as in bladder cancers with *ERCC2* mutations (142) and in cancers associated with tobacco smoking (38). Consequently, the exact molecular mechanism underlying mutations from SBS5 remain unknown (6).

While assigning an etiology to a mutational signature can often rely on associations with specific exposures, the integration of DNA adductomics, comprising of all DNA adduct types and locations, offers an exciting opportunity for a more rigorous approach to hypothesis testing of proposed etiologies. The origins of mutations from different mutational signatures are often complex and multifaceted. These can include mixtures of exposures (e.g. tobacco smoke), broad mechanisms (e.g. DNA repair defects, direct versus indirect effects of UV radiation and chemotherapy), multiple potential sources (e.g. ROS) or even unknown causes. In many instances, adductomics can provide greater clarity by identifying specific DNA modifications, enabling the determination of the reactive species or electrophiles involved and ultimately linking them to the underlying endogenous or exogenous processes. Adductomic data provide direct insights into DNA damage events, enabling a deeper understanding of the mechanisms behind mutational processes. This approach will enhance the strength of etiology assignments, as highlighted by COSMIC’s hierarchy of evidence, where experimental reproduction stands as the highest level of support. Thus, adductomics could serve as a valuable tool to refine and validate etiological links, particularly for signatures without currently proposed origins. Future research should expand current DNA adductomics efforts to support the characterization of mutational landscapes, as part of embracing novel approaches and technologies.

 

#### Site-specific DNA-mutation associations

Lastly, while mutational signatures are largely based on six substitution types, there are actually hundreds of DNA adducts, meaning multiple adducts can give rise to the same mutations. This redundancy could result in a lack of specificity and will likely hinder the identification of the specific key agents in complex environments that are responsible for the actual mutations. Novel technologies, such as nanopore sequencing and others capable of site-specific quantification and mapping of DNA adducts and mutations, are expected to significantly improve our understanding of the precise causal relationships between DNA adduct landscapes and mutational signatures.

#### Filling the timeline gap

Establishing a causal link between exposure-derived DNA adduct landscapes and subsequent mutational signatures necessitates synchronizing data collection at different time stages to simultaneously capture DNA adductomics information and somatic mutations. Presently, DNA adductomics datasets are acquired after acute or chronic exposures, whereas mutational signatures are typically limited to those obtained after a lenghthy selection period. The inherent difference in the time course of the mode of action is highly likely to confound efforts to identify the origin of mutational signatures and may explain why the etiology of most mutational signatures remains unknown. Some exposures lead to a higher mutation burden, while others may not significantly increase the mutation frequency (harder to detect). However, these exposures can alter selective pressures, causing certain clones to die off or creating an environment that favors the expansion of clones with cancer driving mutations. Therefore, future mutation analyses should establish the time course of mutation frequency and types throughout the exposure and selection period, including assessments at the beginning of exposure, to capture mutations that may be lost due to cell death.

#### Embracing innovative technologies

New technologies are constantly evolving, and currently, single-molecule sequencing technologies such as nanopore-based sequencing have shown promise for genome-wide mapping of DNA modification types and locations. In addition, when applied with sufficient read depth, they are, by default, able to determine site-specific mutation frequencies (143). By the nature of this type of sequencing, all these data are obtained on a single molecule and therefore on a single-cell basis.

However, there is a prevailing reluctance stemming from the belief that these methods exhibit significant error rates. Even though manufacturers strive to minimize errors, and some products aim to generate error-free sequences, generating completely error-free data is inherently impossible due to the dynamic nature of genomic DNA, which undergoes constant chemical modification. Moreover, many of the ‘errors’ in base-calling are due to attempts to force a four-letter alphabet (GATC) on a system with more than four ‘letters’ (e.g. GATC plus 5-Me-Cyt, 5-hMe-Cyt, 3-Me-Ade, etc.). Thus, rather than force unreasonable perfection, new developments should extend the sequence approach and base-calling algorithms to include the multitude of known DNA modifications (adducts and epigenetic). This will obviously require a multidisciplinary team and tremendous efforts. Methods for doing this are already being developed – for example, now it is possible to read the ‘six nucleobases’ of DNA at once (144,145). General methods are being developed for detecting DNA modifications (121,146). The huge amount of work ahead should not limit us to improving our understanding of the DNA genome, including its dynamic remodeling. With this, or other emerging technologies, we will significantly advance our understanding of the mechanisms by which the exposome generates a complex DNA adductome and induces the mutational signatures observed in tumors.

Similarly, the approach to DNA adductomics is evolving to being truly a comprehensive assessment of DNA modifications and, as a result, is requiring the development of novel software to analyze the increasingly complex datasets (147). Currently, the focus of DNA adductomics is DNA nucleobase monoadducts, effectively excluding the analysis of more ‘exotic’ adducts. Of relevance to the mutational landscape, newer iterations of DNA adductomics offer the potential to study apurinic/apyrimidinic sites (148), and the advent of nucleic acid adductomics offers the opportunity to encompass DNA–DNA, DNA–RNA and DNA–protein crosslinks (92).

#### Integration of current databases

Even if perfect datasets become available, an unmet challenge will be to establish links between the DNA adductome, the location of DNA adducts and mutational signatures (Figure 1). Currently, to the best of our knowledge, there are no available or suitably applicable (statistical) tools or computational approaches that allow unifying these three fields. Looking forward, the integration of AI, particularly deep learning, could help integrate DNA adductomics, locations and mutational signatures (149,150). This could be combined along with sophisticated bioinformatics tools such as multi-omics data integration platforms for use with comprehensive databases like The Cancer Genome Atlas, Genomic Data Commons, DNA adductome databases (151–155) and exposome-related databases (156–160). Together, this approach will be essential for developing innovative methods to uncover the underlying causes of somatic and mutational signatures by examining the complex nature of the exposome through adductomics (92). These tools should not only facilitate the aggregation of diverse data types, from genomic to environmental data, but also enhance the analytical capabilities necessary to derive meaningful insights from vast datasets. Such a comprehensive approach promises to revolutionize our understanding of cancer etiology and pave the way for precision oncology. By harnessing these advanced technologies, researchers can develop more effective diagnostic tools and targeted therapies, potentially transforming cancer treatment and prevention strategies.

In principle, DNA adductome data can be utilized to estimate mutation probabilities for each adducted or modified nucleotide (G, C, A and T) in a given sequence context. For instance, *N*^2^-BPDE-dG and 8-oxodG adducts predominantly induce G:C to T:A transversions, contributing significantly to the C>A substitutions in mutational signature analyses (Figure 2) (112,161). Site-directed mutagenesis studies employing various TLS polymerases provide crucial information on how the trinucleotide context influences mutation probability (33).

DNA adduct mapping techniques subsequently reveal the tissue-specific distribution of adducts (113,114), allowing for a more precise determination of mutation probabilities within specific sequence contexts. These data are then adjusted to account for dose-response relationships, exposure duration and tissue-specific DNA repair capacities. The resulting comprehensive datasets can be correlated with observed somatic mutation patterns to validate and refine our understanding of mutational processes.

## Conclusion

In conclusion, prior work has provided a vast amount of knowledge about exposures causing cancer and cancer-specific mutational signatures that may retain information about the chemicals causing them. Assembling a comprehensive understanding of the mechanisms from exposure to carcinogenesis will require a concerted effort to integrate diverse data types and to develop new and improved methods capable of unambiguously and simultaneously measuring the landscapes of both exposure and effect, along with mutational signatures.

## Supplementary Material

gkae1303_Supplemental_File

## Data Availability

No new data were generated or analyzed in support of this research.
