# Correction to ‘Investigating the origins of the mutational signatures in cancer’

**DOI:** 10.1093/nar/gkaf1149

**Published:** 2025-10-29

This is a correction to: Gunnar Boysen, Ludmil B Alexandrov, Raheleh Rahbari, Intawat Nookaew, Dave Ussery, Mu-Rong Chao, Chiung-Wen Hu, Marcus S Cooke, Investigating the origins of the mutational signatures in cancer, *Nucleic Acids Research*, Volume 53, Issue 1, 13 January 2025, https://doi.org/10.1093/nar/gkae1303

Shortly after article publication, the authors acknowledged inadvertently omitting Conflict of Interest disclosures for any co-authors. The published article has been updated.

